# Curcumin a Natural Phenol and Its Therapeutic Role in Cancer and Photodynamic Therapy: A Review

**DOI:** 10.3390/pharmaceutics15020639

**Published:** 2023-02-14

**Authors:** Glory Kah, Rahul Chandran, Heidi Abrahamse

**Affiliations:** Laser Research Centre, Faculty of Health Sciences, University of Johannesburg, Johannesburg 2028, South Africa

**Keywords:** curcumin, bioavailability, cancer therapy, photodynamic therapy, natural photosensitize, mechanisms of action, nanotechnology

## Abstract

Cancer continues to cause an alarming number of deaths globally, and its burden on the health system is significant. Though different conventional therapeutic procedures are exploited for cancer treatment, the prevalence and death rates remain elevated. These, therefore, insinuate that novel and more efficient treatment procedures are needed for cancer. Curcumin, a bioactive, natural, phenolic compound isolated from the rhizome of the herbaceous plant turmeric, is receiving great interest for its exciting and broad pharmacological properties. Curcumin presents anticancer therapeutic capacities and can be utilized as a photosensitizing drug in cancer photodynamic therapy (PDT). Nonetheless, curcumin′s poor bioavailability and related pharmacokinetics limit its clinical utility in cancer treatment. This review looks at the physical and chemical properties, bioavailability, and safety of curcumin, while focusing on curcumin as an agent in cancer therapy and as a photosensitizer in cancer PDT. The possible mechanisms and cellular targets of curcumin in cancer therapy and PDT are highlighted. Furthermore, recent improvements in curcumin’s bioavailability in cancer therapy using nanoformulations and delivery systems are presented.

## 1. Introduction

Cancer remains a great threat to human health globally. It is approximated that 20 million cases of cancer were registered in 2020. Despite the considerable advancements in therapeutic methods for cancer, its incidence and mortality have not decreased in the last three decades. It is estimated that 13 million deaths from cancer will be recorded by 2030 [1,2]. Furthermore, an enormous economic burden is shouldered by society and family members of most cancer patients [3]. About 30 to 50% of cancer cases could be prevented if strategies for prevention are implemented and if the predisposing risk factors are minimized. The burden shouldered due to cancer may be minimized via early diagnosis and appropriate treatment [4]. The conventional therapeutic procedures used for cancer and its related diseases include radiotherapy, chemotherapy, and surgery. These procedures frequently cause numerous complications linked to the impairment of healthy tissues. Patients commonly experience a relapse, or the tumor progresses to secondary diseases via metastasis [5,6]. The inability of the conventional procedures to selectively act on malignant tumors motivates the pursuit of more innovative treatments for cancer [6]. Despite the enormous progress made by scientists regarding the discovery of novel bioactive molecules, researchers are continuously seeking to expand knowledge on the potentialities and possible mechanisms of natural products [7,8]. Natural products have been exploited as folklore medicines for years and are confirmed as potential sources from which novel compounds for developing new drugs can be derived [9,10]. Likewise, there is a steady shifting towards different natural compounds, as substantial aftereffects are linked with synthetic chemotherapeutic medications [7,9]. Nature is noted to be a valuable and economic source for new drug discovery and development, and a sustainable environment requires green procedures. Modern analytical methods now allow for bioactive molecules in natural products to be separated, purified, and characterized [9]. The sequencing of the genomes of both pathogens and humans has also tremendously improved knowledge regarding new molecular targets, and this has dramatically elevated the need for new chemical entities (NCEs), also termed new active substances (NASs). Thus, natural molecules are good candidates for new drug development and should be prioritized and invested in by pharmaceutical companies to better understand their mechanisms of action and tap into the advantages that nature itself offers [11].

Curcumin is a natural molecule and the principal active component in the *Curcuma longa L.* rhizomes [12]. A broad range of exciting therapeutic and pharmacological properties have been documented for curcumin, such as antiviral, antibacterial [13], antiamyloid, thrombo-suppressive, chemotherapeutic, antiarthritic, chemopreventive [14], antioxidation, anti-inflammatory, and anticancer, and with few side effects [15,16]. For instance, an in vitro study that exploited about 100 different strains of both gram-positive and gram-negative pathogenic bacteria suggests that curcumin is a good antibacterial agent [17]. Additionally, clinical evidence established that curcumin can be used as a photosensitizer in the treatment of mouth pathogen microbes [18]. Furthermore, scientists have developed an interest in curcumin due to its photokilling and photobiological abilities [15,16], and curcumin appears to satisfactorily meet the needed prerequisites for a promising photosensitizer for PDT [19]. Mindful of the pharmacological properties of curcumin aforementioned, particularly regarding cancer treatment, it has not yet received approval for cancer clinical therapies. Since cancer remains the leading cause of human death globally, demand for more effective therapeutic procedures using anticancer agents that could induce less toxicity is imperative. This review thus focuses on curcumin and its potential in cancer therapy and cancer PDT, and how nanotechnology can ameliorate the efficacy of curcumin in cancer PDT.

## 2. Source and Discovery of Curcumin

Curcumin is a natural compound isolated from the rhizome of the turmeric (*Curcuma longa Linn)* plant belonging to the ginger (*Zingiberaceae)* family [20,21]. The plant is stated to have originated in India, Indonesia, and Southeast Asia and is now cultivated globally in subtropical and tropical areas [22]. Turmeric powder is popularly called “Golden spice” due to its bright orange-yellow color and has been extensively used as a spice (a flavoring and coloring agent for curries) and in mustards [20,23]. Traditionally, turmeric has been used for various purposes as folklore medicine, and its medicinal effects have been cited in Unani, Ayurveda, Chinese, and Siddha medicine. This has led to its reputation for therapeutic effects [24,25]. Turmeric is a popular known medicinal herb and has broad pharmacological activities, including hypocholesterolemic, antidiabetic, antithrombotic, antidiarrheal, carminative, antihepatotoxic, diuretic, antirheumatic, hypotensive, larvicidal, antiviral, antityrosinase, insecticidal [26], antioxidant, antimicrobial, antiprotozoal, anti-inflammatory, antivenom activities, antiaging, antiangiogenic, antiproliferative, antimalarial, and antitumor properties [22]. Furthermore, it can be utilized to treat parasitic infections, ulcers, symptoms of flu and colds, immune diseases, and skin diseases [23].

However, the chemical constituents and pharmacological effects of turmeric are mostly attributed to its curcuminoids content, mainly comprising about 77% curcumin and 2 related derivatives, namely bisdemethoxycurcumin (BDMC) and demethoxycurcumin (DMC), with a percentage composition of about 3% and 17%, respectively [22,27]. In 1815, a yellow substance was isolated from turmeric by Vogel and Pelletier; this was called curcumin and was identified as the most abundant molecule in it [26]. Subsequently, in 1910, the chemical structure of curcumin was established as diferuloylmethane by Milobedzka and team, and in 1913, they produced a synthetic version of curcumin [28,29].

## 3. Photophysical Properties of Curcumin

Curcuminoids that are bisdemethoxycurcumin, demethoxycurcumin, and curcumin are naturally hydrophobic. The main biological activity of curcuminoids is linked to their active ketone, methoxy, and phenol functional groups, which are vital in promoting apoptosis and antioxidation [30,31]. Curcumin can boost various biochemical reactions via hydrogen donation to cause oxidation, irreversible and reversible nucleophilic reactions (Michael reaction), hydrolysis, and enzymatic and hydrolysis reactions [32]. Nonetheless, curcumin is a naturally occurring photochemical substance with a broad spectrum of absorption (300–500 nm) [18]. Furthermore, the maximum absorption wavenumber for curcumin can vary depending on its solvent systems [33]. Curcumin can be photo-excited within a very small timescale (sub-nanosecond) and with atoms of hydrogen being transferred. This transfer of atoms of hydrogen occurs in an ultrafast manner, and the associated changes in conformation can be vital to the overall pharmacological characteristics of curcumin [11]. The maximum fluorescence excitation wavenumber is 425 nm, while the maximum emission wavelength is 530 nm, and this can stimulate very strong phototoxicity, even at very small (micromolar) concentrations [33]. This phototoxic effect of curcumin forms the basis for curcumin exploration in PDT. Still, knowledge of curcumin concerning its solubility, log P, dark toxicity, stability, photobleaching, chemical structure, and optical characteristics is essential in the proper elucidation of its pharmacodynamic and pharmacokinetic potentials in PDT protocols. Table 1 presents the key chemical and physical properties known for curcumin.

## 4. Safety and Bioavailability/Pharmacokinetics of Curcumin

Curcumin and other turmeric-derived products are labeled as safe by the Food and Drug Administration (FDA, USA), and curcumin has achieved therapeutic pursuit in treating metabolic diseases, immune-related diseases, and cancer, owing to its vast biological target and with practically no aftereffects [39]. The structural characteristics of curcumin improve its ability to bind with various biomacromolecules. Curcumin vital biochemical reactions are achieved via a H-bond in the β-dicarbonyl group, two methoxy group residues, and via the phenolic hydroxyl residues, by bonding to non-metals and metal [29]. Curcumin can also directly bind to ribonucleic acid (RNA), deoxyribonucleic acid (DNA) [40], and various signaling molecules, including protein reductase, protein kinase, inflammatory molecules, histone deacetylase, histone acetyltransferase, xanthine oxidase, glyoxalase I, (human immunodeficiency virus) HIV-1 protease, HIV-1 integrase, DNA methyltransferase-1, calcium ATPase in the endo/sarcoplasmic reticulum, metal ions, and carrier proteins. The bonding of the diketo functional group in curcumin with transition metals forms chelates, decreasing the induced toxicity of the metal. Meanwhile, an augmented antioxidant activity will be triggered by part of the metallic complexes since they mimic enzymatic properties [36]. The binding of curcumin with carrier proteins enhances both its bioavailability and solubility. Curcumin is often not stable at physiological pH. It quickly degrades via autoxidation to bicyclopentadione as the major product, where the 7-carbon chain undergoes double cyclization and an oxygenation reaction [40]. The bicyclopentadione oxidation products and products from alkaline hydrolysis, such as feruloylmethane, ferulaldehyde, ferulic acid, and vanillin, exhibit less bioactivities than curcumin [29].

However, curcumin′s metabolism greatly hinders its clinical applications. The utilization of curcumin is severely hampered by its poor bioavailability caused by its poor absorption in the small intestine; low tissue and low plasma thresholds; rapid hepatic metabolism, fast systemic excretion, and body clearance; inactive metabolic products; and low intrinsic activity [29,41,42]. Reports from bioavailability and pharmacokinetic studies attest that curcumin’s intestinal absorption following oral administration is extremely low, and its elimination from the body is very rapid [41]. Often, oral intake of curcumin is eliminated via feces without being metabolized, and only a small amount is absorbed and modified during metabolism [43]. A study using rats indicated that oral administration of curcumin at 1 g/kg dose was mostly eliminated via feces (75%), and just a little proportion was absorbed in the intestines [44]. In the hepatocytes and enterocytes, reductases catalyze the bioreduction of curcumin, and dihydrocurcumin, tetrahydrocurcumin, hexahydrocurcumin, and hexahydrocurcuminol (octahydrocurcumin) are generated as reduced products [43]. These reductases may include alcohol dehydrogenase, nicotinamide adenine dinucleotide phosphate (NADPH)-dependent reductase, and a microsomal unidentified enzyme [45]. Moreover, Hassaninasab et al. [46] purified and characterized an enzyme from *Escherichia coli* that was used in reducing curcumin. It was discovered that curcumin’s microbial metabolism with the purified enzyme involves a two-step reduction that depends on NADPH, where curcumin is first converted to dihydrocurcumin, the intermediate product, and then to tetrahydrocurcumin, the final reduction product [46]. By assessing the metabolic path of curcumin in humans and rodents, it was observed that its absorption is linked with reactions, including glucuronidation and sulfation, which occur in various tissues [44,47]. Reduced derived metabolites of curcumin and curcumin itself are easily conjugated into sulfate and glucuronic acid, both in vitro and in vivo. The intestines and liver of humans and rats are the principal sites for curcumin′s sulfation and glucuronidation reactions, and these reactions are accelerated by sulfotransferase and glucuronyl transferase, respectively [43]. The liver is considered a major organ liable for curcumin’s fast metabolism [42]. Yet, curcumin binds to the human intestinal epithelium at a very fast rate compared to hepatic microsomes, and this is contradictory to what happens in rats [48].

Curcumin also has poor solubility in water (estimated at 11 ng/mL at pH 5.0), and this hinders its intestinal absorption, which could further cause low bioavailability. Nonetheless, following oral intake of curcumin by humans, part of it is absorbed as water-soluble sulfate and glucuronide conjugate in plasma. The human phenol sulfotransferases (SULT1A1 and SULT1A3) are accountable for curcumin sulfation in human and rat intestines, whereas the glucuronidation is catalyzed by uridine diphosphate-glucuronosyltransferase (UGT). Conjugation or reduction of curcumin leads to the production of molecules that have a reduced potential to inhibit cyclooxygenase-2 (COX-2) expression when compared to curcumin. Likewise, curcumin sulfate, hexahydrocurcumin, and tetrahydrocurcumin exhibit weaker inhibitory effects on prostaglandin E2, and the hexahydrocurcuminol remains inactive [49]. Similarly, curcumin metabolites in humans’ and rats’ bile are often hexahydrocurcumin and tetrahydrocurcumin glucuronides, and with minor metabolites, including dihydroferulic and ferulic acid, that combine with bile salt and are then rapidly metabolized for elimination by the body [50]. The bio-activities of most curcumin-derived metabolites, except for tetrahydrocurcumin, are drastically decreased compared to curcumin [30,51].

## 5. Anticancer Therapeutic Effects of Curcumin

Tremendous advancements have been made over the past decades to understand the molecular biology involved in the evolution of cancer. Still, the dreaded disease remains unconquered [52]. Curcumin is an extremely pleiotropic molecule that can mediate cancer treatment as a chemopreventive or chemotherapy agent without side effects [53]. Natural compounds are now mostly considered in the search for molecules to be used in cancer intervention. Compounds extracted from medicinal plants and dietary sources are validated for having considerable chemopreventive properties [54]. Curcumin is an example of such a compound and is admitted to interference within the multi-stages of carcinogenesis [55]. Chemoprevention is now a rapidly growing domain in anticancer studies, with the spotlight on different interventions, including nutritional, pharmacological, and biological factors [56]. The word “chemoprevention” was originally defined in 1976 by Sporn M. B. as a preventive modality where synthetic or natural agents could be utilized to prevent, reverse, or slow the progression of cancer [57]. The purpose of chemoprevention compounds is to interpose in the precancerous initial phase of carcinogenesis before the manifestation of malignancy, minimize the risk of cancer at the early stage, and avert the spread of secondary tumors [52,58,59]. Furthermore, the agents’ toxicity may influence patient accrual when employed in large-scale real clinical studies. Compared with synthetic compounds, the natural compounds from plant sources [vegetables, species (curcumin), and fruits] are well known for their safety and have been historically consumed by humans for a long period [60]. However, poor diet and nutrient factors are significantly associated with susceptibility to various cancers developing [56]. Approximately 35% of cancer mortality worldwide is linked directly to dietary-incited factors [55]. Dietary-derived polyphenolic compounds such as curcumin are stated to have anticancer potential and related pharmacological benefits and receive enormous attraction because of their beneficial health impact [61,62]. Selecting natural compounds for the prevention of cancer may be an effective and justifiable strategy for individuals with an elevated risk of developing cancer.

Three principal approaches are implicated in the chemoprevention of cancer, which is categorized into primary, secondary, and tertiary preventive approaches. The primary interventions are those envisioned to prevent normal healthy persons from developing risk factors, such as preventing predisposing genetic factors to avert the development of some cancers. The secondary approach was developed to avoid cancer progression by treating premalignant tumors. The third preventive approach is to treat cases with a known cancer history, hence preventing any further relapse or progression to primary [54]. Chemoprevention agents were categorized in 1985 by Wattenberg into inhibitors of cancer-forming agents (carcinogens), blocking agents, and suppressing agents [63]. These agents help to prevent the formation of cancer from its precursors, and the blocking agents prevent carcinogens from stimulating mutagenic effects by specifically blocking the pathways involved in the activation of metabolic carcinogens. They equally boost the process of detoxification by trapping certain types of reactive oxygen species (ROSs). The suppressing agents then prevent the progression of tumors by blocking the proliferation of cells and their differentiation, induced apoptosis, necrosis, and autophagy [58,59]. Curcumin similarly exhibits various chemopreventive molecular mechanisms, such as molecular signal inhibition, apoptosis induction, inflammatory response inhibition in the tumor microenvironment, and free radical scavenging. Curcumin ideally presents good the features of a chemoprevention agent, such as being easily accessible, cost-effective and low toxicity, and having an anticancer, multitargeted effect [53,56,61]. Mindful of curcumin′s chemopreventive ability, it also has chemotherapy potential. Chemotherapy is a treatment method for cancer. It is described as the exploration of different drug types to kill cancerous cells via various mechanisms. Normal cells can be equally affected by chemotherapy [64]. However, the chemotherapeutic effect of curcumin on normal cells has been demonstrated to be different. Curcumin cellular uptake by normal cells is lower than in cancerous cells [65]. Normal cells are noted to have higher levels of glutathione than cancerous cells, thus decreasing the sensitivity of normal cells to curcumin. Furthermore, normal cells do not actively express nuclear factor-kappaB (NF-κB), as in the case of cancerous cells, where the NF-κB is constitutively expressed and its survival is also mediated. Moreover, curcumin has shown no biological effect on normal hepatocyte cells in rats, generating no superoxide and, thus, no death of cells [66,67]. Notwithstanding, if the approach to treating cancer is via a chemotherapeutic or chemopreventive agent, multiple targets and modulation pathways are needed in cancer therapy due to its multifactorial nature [68]. Different molecular targets involved in chemotherapy could be relevant in cancer chemoprevention, and curcumin is documented to interfere in various molecular mechanisms during therapy [69].

### Therapeutic Mechanisms of Curcumin in Cancer

Curcumin′s anticancer ability is known to be one of its crucial effects, and different molecular targets are being obstructed [70]. Nonetheless, the principal leading causes of cancer are the deficiencies in balancing cell proliferation and cell death. The skipping of cell death contributed by non-apoptotic signals and unrestrained cell proliferation gives rise to various forms of cancers [71]. Apoptotic signals induced by curcumin are generated using intrinsic and extrinsic pathways. Mitochondrial membranes are triggered in the intrinsic pathway to hinder the antiapoptotic proteins, B-cell lymphoma extra-large (Bcl-Xl) and B-cell lymphoma-2 (Bcl-2), from expressing [72]. The potential balance in the mitochondrial membrane can be disrupted by curcumin, boosting the suppression of Bcl-xL protein [73]. In the extrinsic pathway, apoptosis is stimulated by multiplying the receptors for cell death and activating related apoptosis via tumor necrosis factors (TNFs). Equally, in this pathway, curcumin can upregulate death receptors (DR-4 and DR-5) expression [74,75,76]. It is reported that the administration of 12 g/day of curcumin for a duration of 3 months in patients can induce antiproliferative and apoptosis responses against various cancers, including colorectal, prostate, kidney, breast, and pancreatic cancer [77,78]. In a study using mice, various doses of curcumin at 0.1 to 3 mg/kg of body weight in mice were said to suppress the reverse transcriptase telomerase enzyme [77] and decrease the expression of Bcl-2 [79]. Findings from in vitro experiments disclosed remarkable effects of curcumin and its derivatives in inciting apoptosis in various cell lines via downregulation or inhibition of cell transcription intracellular factors. The factors inhibited were activator protein 1 (AP-1), signal transducer and activator of transcription-3 (STAT3), nitric oxide synthase, COX-2, matrix metalloproteinase-9 (MMP-9), and NF-κB [80,81]. Another in vitro study presented a very novel mechanism of curcumin pyruvate kinase M2 (PKM2) downregulation in cancerous cells via the Warburg effect (decreasing lactate production and uptake of glucose). PKM2 inhibition was accomplished by suppressing rapamycin-hypoxia-inducible-factor 1α (TOR-HIF1α) in the targeted mammalian [82]. However, the anticancer ability of curcumin is largely mediated via the opposing effects on different transcription factors, protein kinases, inflammatory cytokines, growth factors, and various oncogenic molecules. Likewise, its potential to increasingly suppress growth factors, including TP53, Rb, and p57Kip2, as well as downturn-related proliferation pathways, such as phosphatidylinositol-3 kinase (PI3K), Janus kinase-(JAK)-STAT, wingless-related site (Wnt)-Beta-catenin, Sonic Hedge-hog, activator protein 1 (AP1), mitogen-activated protein kinases (MAPK), transforming growth factor-beta (TGF-β), and NF-κB, acts against angiogenesis [angiopoietin and vascular endothelial growth factor (VEGF)] and enhances apoptosis in various cancers [69].

In addition to the aforementioned molecular targets, curcumin can also obstruct the micro-ribonucleic acids (miRNAs). The miRNA (small non-coding RNA) is vital for the cell’s physiological conditions, such as apoptosis, growth, differentiation, and angiogenesis [83,84]. Alterations in these conditions can either downregulate or upregulate various molecular and cellular targets involved in cancer progression [85]. Likewise, in vitro evidence from different sources indicates that curcumin could exercise its anticancer ability by upsetting the expression of miRNAs, such as miR-208, miR-21, miR-9, miR203, miR-181b, and miR-19 [86,87,88]. The Jin et al. [89] in vitro study with molar concentrations of curcumin (5 to 40 μM) relative to miRNA thresholds showed that 10 and 20 μM of curcumin upregulated miR-192-5p in non-small cell lung cancerous cells via the modulation of the PI3K/protein kinase B (Akt) signaling pathway. Similarly, the upregulation of miR-21 with 50 μM of curcumin was noted in thyroid carcinoma [90]. Findings from in vitro studies also affirm that curcumin can upregulate the enhancer of zeste homolog 2 (EZH2) breast cancerous cells and downregulate deletion gene 1(DLC1) in hepatocellular carcinoma. The inhibition of EZH2 expression helped in restoring DLC1 expression; hindered invasion, migration, and proliferation; obstructed the cell cycle; and enhanced apoptosis in MD Anderson metastatic cancer (MDA-MB-231) [91].

## 6. Photodynamic Therapy (PDT)

PDT is an advancing nonconventional medical modality for the treatment of non-oncological and oncological illnesses via the integration of light, drugs (photosensitizer), and oxygen. The modality is non-invasive and can eradicate diseased cells, whereas healthy tissues are left unharmed. These qualities make PDT a more ideal modality in the cancer therapeutic approach compared to conventional modalities, such as radiotherapy, surgery, and chemotherapy, as they have been linked with limited efficiency and numerous aftereffects [92,93]. It was later established that oxygen from the air was equally essential for the mediated killing of microorganisms by light, and the phenomenon was then coined “photodynamic action”. The first effort to explore this phenomenon for cancer treatment was done by covering the superficial skin tumor layer with dyes and then exposing the area to light [94].

In the 1970s, the modern era for PDT was initiated, greatly due to work by Dr. Thomas Dougherty and co-workers at the Buffalo, New York, NY, USA, institute for cancer (Roswell Park Cancer Institute). They introduced the first photosensitizer (PS) for PDT. This PS was designated as a hematoporphyrin derivative, which is a water-soluble solution of porphyrins. Subsequently, photofrin was obtained as a purer form of this PS. Photofrin is the most utilized PS at present. However, it is recognized to have disadvantages, such as skin photosensitivity, which may last many weeks, and demonstrates low absorbance at 630 nm [95]. Nonetheless, effort has been made by medical chemists to produce or discover molecules with improved PS properties, and different compounds have been proposed for potential utilization to mediate PDT for treating cancer and several other diseases [96].

### 6.1. Mechanism of PSs in PDT

Type I and II photochemical mechanisms can be needed for a PS agent or drug to induce cell death in PDT [96]. The PS at the ground state appears in singlet form and has double electrons with spins in opposite directions. The spin sum total is zero, hence, denoted with the symbol S0 (exemption of oxygen molecular). The absorption of an appropriate wave number (quantum energy) of light by PS will provoke an electron excitation to an orbital with higher energy (PS I). The unstable state of singlet PS may lead to it losing its excess of energy by the production of heat via internal conversion or via emitting light (process of fluorescence). The PS in its excited singlet state can still undergo an intersystem crossing (IC) to form a steadier triplet-excited state having parallel spins (PS II). At this state, the angular momentum for the spin with two unpaired electrons adds to the quantum spin of number 1. Moreover, at this step, the excited PS at its triplet state can decay back to its ground state via the emission of a phosphorescent photon. However, by conferring with the rules of quantum selection, this process can be considered forbidden. The PS at the triplet state is more stable than when in its singlet state and has a longer lifetime (microseconds), as compared to its singlet excited state with a shorter lifetime (nanoseconds). A photodynamic type I reaction or mechanism can materialize by reactions that involve electron transfer to produce ROS, including the superoxide radical anion (O_2_^•−^), hydroxyl radicals (HO^•^), and hydrogen peroxide (H_2_O_2_). Furthermore, a type II reaction can then occur where the triplet state PS with its longer lifetime transfers energy to molecular O_2_ to form singlet oxygen (^1^O_2_). The ^1^O_2_ and ROS generated can trigger oxidative stress in biomolecules, commonly in pathogenic and cancerous cells. The biomolecules targeted can include proteins, lipids, nucleic acids, and various other biomolecules within the tumor [97,98]. Cell death via apoptosis is mostly triggered when the singlet oxygen or cytotoxic ROS that was generated via the PS impairs the cell’s mitochondria; but if the cell membrane is impaired, cell death by necrosis will happen, as the loss of cellular integrity will occur. When only the endoplasmic reticulum or lysosome functions are impaired, then cell death will mostly arise via autophagy [99]. However, the ROS produced in Type II photochemical reactions is noted to be mechanically simpler than through the Type I mechanism, and PSs in anticancer PDT are often believed to function by the Type II mechanism instead of Type I [96].

### 6.2. Photosensitizers

PSs are defined based on their chromophore contents, which are established as unsaturated conjugated bonds able to absorb a particular wavelength of light with an elevated absorption coefficient. Selecting a suitable PS for PDT is extremely crucial since the efficacy of PDT greatly depends on the PS properties [100]. Thus, for a PS to be exploited for cancer treatment and other diseases, it should ideally meet numerous conditions: (1) The PS should not exhibit toxicity in the dark; (2) The PS should be obtained easily as a pure compound, and its chemically attributed properties must have been established judiciously in previous literature; (3) The PS should have an elevated absorption coefficient, with a spectral range from 600 to 800 nm and with maximum penetration of light via tissue; (4) In aqueous solvents, the PS should be stable and soluble; (5) The PS should generate a high quantum yield at the triplet state, singlet oxygen, and ROS properties for optimal PDT sensitizers [96]; (6) The PS should be absorbed selectively in a specific target tissue; (7) The PS should be rapidly eliminated from the body to avoid phototoxicity; (8) The PS should be able to selectively bind to intracellular sites with high susceptibility to oxidative damage; (9) The PS should present optimum pharmacokinetic qualities [101]; (10) No toxic impact should be induced by the PS on healthy tissue; and (11) The PS (drug) should be able to be manipulated within short, light intervals to ease treatment for outpatients.

However, no proposed PS has perfectly embodied the features of an ideal PS, and efforts to develop new and more effective PSs with optimal properties for PDT continue [102,103]. Curcumin (photosensitizer) is mentioned as a newcomer in PDT, although it has been recognized as a medicinal compound and spice for centuries [13,96]. Current studies of curcumin in PDT have shown its curative anticancer effects [104,105]. Low concentrations of curcumin in combination with visible light or ultraviolet A (UVA) irradiation were exploited for the first time by Dujic et al. [106] to activate anticancer activity. More recent studies have revealed that curcumin as a photosensitizer in PDT can hinder proliferation and profoundly induce apoptosis in various cancer cells, and this means it has great potential for use in cancer treatment [105,107].

### 6.3. Anticancer Effects of Curcumin in PDT

The efficacy of curcumin in PDT has been demonstrated using various types of cancerous cells. The molecular mechanism and apoptotic effects of demethoxycurcumin (the photosensitizer) and UVB radiation combined treatment were studied using skin cancer cells (HaCaT and A431 cells). The results revealed apoptosis in both cell lines, and pathways for caspase and p53 were activated, causing an increased expression of nuclear factor-κB (p-p65) and Bcl-2-associated X protein (Bax), and suppressing the NF-κB pathway, myeloid leukemia-1(Mcl-1) and Bcl-2. ROS was elevated and mitochondrial membrane depolarization was substantial [108]. Buss et al. [109] revealed that incubation of cancerous melanoma cells (A375 and G-361 cells) with 0.2 μg/mL of curcumin for 1 h, followed by visible light (5500 lx) exposure, led to a significant decrease in cell proliferation and viability. Further, the ultraviolet A-irradiated cells were compared to those treated with visible light, and it was proven that visible light induced a greater inhibitory effect on the viability and proliferation of A375 and G-361 cells, without interfering with cell membrane integrity. Cell apoptosis estimated at over 90% was obtained with the optimum treatment combination [109]. Elevated apoptotic nuclei were established in HaCaT cells within 24 h after the cells were incubated for 1 h with 0.1–1 mg/mL of curcumin and exposed to visible light (5500 lx) for 5 min. These treatments induced the release of cytochrome c in HaCat cells and inhibited the pathway for NF-κB, which subsequently activated caspases 8 and 9, leading to apoptosis [106]. However, a Laubach et al. [107] study indicated that utilization of curcumin in PDT to stimulate apoptosis in tumor cells is not dependent on the initial signal ligand for apoptosis and the tumor necrosis factor (factor-α receptor) [107]. A study that examined the cellular light energy uptake of A375 cells via the target excitation of curcumin with light radiation of various wavelengths established that the amalgamation of blue (405 nm) and red (630 nm) light efficiently enhances autophagy, hence inhibiting proliferation [110]. A photodynamic study of two natural photosensitizers (curcumin and hypericin) on skin cancer confirms that curcumin is more suitable for skin cancer treatment [42]. The Shao et al. [105] study on A549 and SPCA1 lung cancer cells indicated that autophagy was induced in these cells after the combined curcumin and PDT treatment. The induction of autophagy was markedly increased 24 h post-PDT, as the expression of beclin1 and LC3-II was increased, while p62 expression decreased. Moreover, increased E-cadherin expression and decreased expression of vimentin, Snail, and N-cadherin were noted, which contributed to enhancing the inhibition of the epithelial–mesenchymal transition (EMT). The curcumin-PDT treatment also alleviated both the migration and invasion of lung cancer cells. Hence, the combined treatment led to the inhibition of EMT, invasion, and migration, whereas autophagy was induced in the cancerous cells [105].

Furthermore, PDT is suggested as a therapy that can better target and ameliorate breast cancer treatment. Current research on curcumin in PDT for breast cancer therapy mostly utilizes curcumin nanocomposites. Nanocomposites of curcumin anionic nanoclay (0 to 100 μg/mL) with blue light were used to treat MDA-MB-231 cells. The study confirmed induced apoptosis, autophagy, antiproliferation, cell cycle arrest at the G0/G1 phase, and increased ROS in MDA-MB-231 cells. Curcumin anionic nanoclay was proposed as a novel method to enhance the PDT for breast cancer [111]. The PDT ability of low curcumin concentration (0.01 to1 μg/mL) was investigated on human oral squamous cell carcinoma cells (HN cells), and a 30% decrease in cell proliferation was noted. The utilization of 0.2 μg/mL of curcumin merged with light resulted in substantial augmentation in DNA fragmentation, which was estimated to be threefold higher than in the control group [112]. Further, curcumin led to a considerable reduction in resistance in colorectal cancer cells (Caco-2 cells), hence, promoting the mediation of the 5-aminolevulinic acid (5-ALA) in PDT. As a result, the Caco-2 treated cells had a significant reduction in viability of about 62.4% [113]. SW620 cells were treated with blue laser light (470 nm), resulting in cell viability inhibition, reduced proliferation and migration, and increased cell death, which could have been triggered by the increased accumulation of ROS and DNA damage [114]

### 6.4. Possible Anticancer Mechanism of Curcumin in PDT

**Figure 1:**
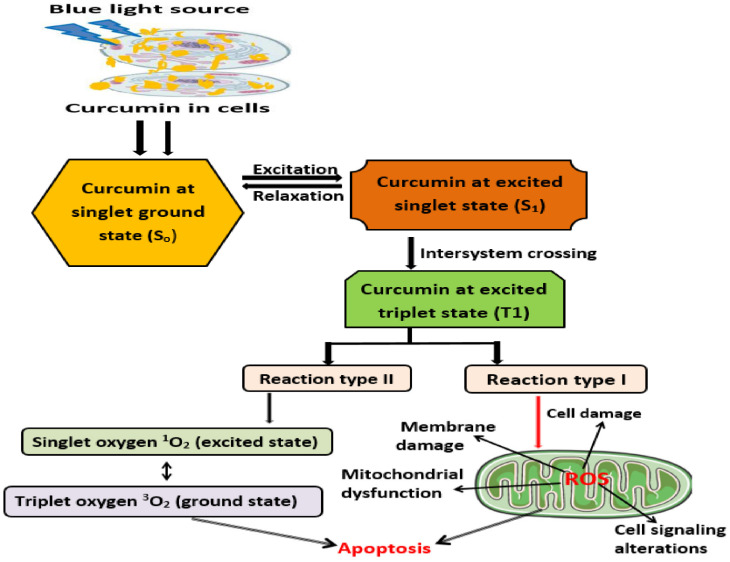
Absorption of specific blue-light irradiation by curcumin in cells can induce its excitation from the S_0_ phase (singlet ground state) to the S_1_ phase (excited singlet state). Curcumin can then enter the T_1_ phase (the excited triplet state) after the intersystem crossing. Two types of reactions (type I and type II reactions) may occur at the T1 phase. Type I reactions involve reactions with biological molecules, and free radicals are formed via the transfer of electrons. ROS is generated via reactions of free radicals with oxygen. In a type II reaction, oxygen can directly react with curcumin (PS) in the excited triplet state to produce singlet oxygen. ROS production and apoptosis induction caused by curcumin photosensitization can cause various types of cell damage.

Naturally occurring photosensitizers, such as curcumin, can induce a photodynamic impact at the targeted site with cells via its activation by light radiation. As illustrated in Figure 1, the absorption of blue-light radiation of a specific wavelength number by curcumin could cause electrons to transition from one state to another. Curcumin at its ground state, S_0_ (singlet state), could be stimulated to its excited state, S_1_, with simultaneous production of corresponding energy. An additional part of the generated energy directs curcumin (photosensitizer molecule) to enter into a steadier triplet state (T1) [11]. At this state, energy is gained by nearby biomolecules, which then transfer electrons or hydrogen ions to form various free radicals. Subsequently, they can react with oxygen to generate ROS [115]. The accumulation of ROS to a particular threshold incites oxidative stress, with reactions leading to direct cell damage, blood vessel indirect closure, and immune pathway activation, inducing non-programmed and programmed pathways (necrosis and apoptosis) to trigger cell death [96]. The production of ROS resulting from curcumin in PDT has been reported in different in vitro studies that exploited cancerous cell lines. Combined treatment with curcumin and visible light has resulted in significant photocytotoxicity in cell lines, such as L929 fibroblasts [116], HeLa cells [117], HN cells [112], SNB19 cells [118], and LNCaP cells [19].

#### 6.4.1. Effect on Mitochondrial Membrane

Photosensitization of curcumin in PDT can lead to the obstruction of mitochondrial function in tumor cells in two ways. Firstly, Bcl-2 can hinder apoptosis by blocking the mitochondria from releasing cytochrome c and downstream and upstream stimulation of caspases. Meanwhile, a heterodimer can be formed by the bonding of Bax with Bcl-2, thus preventing apoptosis inhibition by Bcl-2. The stability and permeability within the mitochondrial membrane could be impacted by curcumin in PDT via augmenting the mediation of calcium ions by Bax and Bcl-2 at the endoplasmic reticulum. Secondly, cathepsin lysosomal enzymes can activate Bid, causing it to alter the mitochondrial permeability and induce cellular death pathways, such as autophagy and apoptosis [42].

#### 6.4.2. Effects on Cell Apoptosis

Curcumin combined with PDT can trigger cell apoptosis via effects such as DNA fragmentation, obstructing the cell cycle, and altering the pathways for apoptosis. DNA fragmentation is described as one of the principal biochemical features of apoptosis. Proliferating or normal cells will hardly show DNA fragmentation. A study revealed that the photosensitization of curcumin in HaCAT, A549, and A431 cells led to corresponding DNA fragment rates of 16, 5, and 3 times, respectively, which were elevated in these experimental groups compared to control groups [106]. Similarly, extensive inhibition of proliferation and elevated DNA fragmentation in HN cells were established after the photosensitization of curcumin in the treated cells [112].

Moreover, curcumin combined with PDT can obstruct the cell cycle to induce apoptosis and equally alter the thresholds of cyclin expression. This combined treatment can induce cyclin expression in cancerous cells through the regulation of NF-κB pathways, thereby mediating the phases involved in the cell cycle. A combination of visible light (5001x, 5 min) and a low concentration of curcumin were noted to block the process of the cell cycle at the G0/G1 phase in renal cancer cells (KTCTL-26, A498, Cakil) [119]. Similar findings on bladder cancer cell lines established that visible light and curcumin combined treatment can block the G0/G1 phase in RT112 cells via the inactivation of cyclin-dependent kinase (cdk), 2-cyclin A, and cdkl-cyclin B [120]. Furthermore, bladder carcinoma cells (UMUC3 and TCCSUP cells) were induced by cdk2-cyclin A/cdkl-cyclin B to enter the G2/M phase due to curcumin-mediated PDT treatments [120,121].

A combination of curcumin and PDT treatment has been described to obstruct apoptosis and related protein pathways [122]. This treatment can activate cell apoptosis via the exogenous and endogenous pathways by regulating caspase proteins, such as caspases 3, 8, and 9, and a variety of other apoptotic pathways [123]. A study by Dujic et al. [106] on HaCAT cells showed an important anticancer mechanism for curcumin treatment in combination with visible light or UVA irradiation. Nuclei staining with bisbenzimide post-treatment revealed marked elevations in apoptotic nuclei. To add clarity to this, cytochrome c expression and caspase activation were examined. Curcumin-mediated PDT induced cytochrome c release from the mitochondria in HaCaT cells and activated caspases 8 and 9. Interestingly, the HaCaT cells showed a time-dependent separation for extrinsic and intrinsic apoptosis via activation of caspases 8 and 9. The markers of apoptosis could be confirmed given that the activation of caspase 8 occurred 30 min after the activation of caspase [106]. Caspase-9 expression in a A431 tumor was activated, and significant decreases in phosphorylation levels for the epidermal growth factor receptor (EGF-R) and extracellular regulated-kinase1/2 (ERK1/2) were noted [124]. It is emphasized that the combined treatment of cells with curcumin and PDT can indirectly regulate apoptosis using the NF-κB and PI3K/Akt signaling pathways, which are involved in regulating the survival and proliferation of cells, thus controlling expression from the Bcl-2 family. Moreover, increases in C-jun N-terminal kinase (JNK) phosphorylation could activate apoptosis via the endogenous pathway and enhance apoptotic factors to be released. The increase in JNK levels of phosphorylation can stimulate the downregulation of Akt levels of phosphorylation. The signaling pathway for the AKT-mammalian target of rapamycin (mTOR) and the protein Bcl-2 antiapoptotic in kidney cancerous cells can be hampered by curcumin plus PDT treatments, while simultaneously increasing Bax expression and causing apoptosis [106,110].

#### 6.4.3. Effects on Cell Viability and Proliferation

A study by Rutz on KTCTL-26 and A498 cells indicated that no increase in cell proliferation was obtained with single treatments of curcumin or visible light only. Treating these cells with both light and curcumin (0.1 to 0.4 µg/mL) induced a significant dose-dependent decrease in cell proliferation, with maximum activity at 0.4 µg/mL [119]. A Dujic et al. [106] study confirmed that primary keratinocytes and HaCaT keratinocytes treatments with low concentrations (0.1–1 µg/mL) of curcumin alone did not influence cell proliferation, as DNA synthesis was unaffected. The combined treatment of these same cells with curcumin (0.2 µg/mL) and visible light led to remarkable results, with DNA synthesis inhibition indicative of cell proliferation inhibition. To validate if the effect of light irradiation could be cell-type specific, various cell types, including melanoma, melanocytes, epidermoid carcinoma, and primary skin fibroblasts, were tested. The curcumin with light treatments was able to inhibit all the cell types from proliferating [106]. A study on nasopharyngeal cancerous cells yielded similar results, and irradiation from blue-filtered light and visible light on curcumin-treated cells improved cytotoxicity [125]. In addition, the photosensitization of SNB19 cells allowed for a 6,3-timerless concentration of curcumin (drug), and the viability of cells was about 50% lower when compared with curcumin treatment without radiation [118].

## 7. Nanotechnology

The exploration of nanotechnology has significantly improved the pharmaceutical sector in recent years via the discovery of new nanoparticles. Extraordinary novelties have been realized via the production and delivery of novel drug active principles using nanotechnology [126]. Furthermore, nanotechnology methods have been exploited to fabricate several nanoformulations of curcumin (nanocurcumin), such as liposomes, polymers, nanoparticles, conjugates, micelles, solid dispersion, cyclodextrins, nanogels, and nanodisk. These nanocurcumins have been developed with the ultimate focus to improve the solubility and bioavailability of curcumin and also shield it from inactivation by hydrolysis. Some of the formulations are envisioned for longer retention and circulation in the body, whereas some are for intracellular release mechanisms and targeted delivery of drugs in specific cells [127]. Most of the nanoformulations of curcumin have created a valuable impact on various pharmaceutical applications and have been documented to ameliorate the anticancer ability of curcumin [127]. A study on melanoma (B16BL6) cells using curcumin-loaded liposome (Cur-Lip) nanoparticles validated that Cur-Lip nanoparticles severely hindered the proliferation of B16BL6 cells. This was chiefly contributed to better delivery of the drug, allowing for the fusion of particles into the cell’s intracellular space. The PI3 K/AKT pathway known to be vital in various types of skin carcinogenesis was inhibited [128]. Chang et al. [129] investigated the mechanisms triggered in CAL27 cisplatin-resistant cancer cells (CAR) by curcumin-loaded poly(lactic-co-glycolic acid) (PLGA) nanoparticles. The proposed data showed that the nanoparticles regulated the activation of multiple-resistance drug-protein 1 and ROS in CAR cells by stimulating intrinsic pathways for apoptosis. Additionally, the curcumin-loaded PLGA nanoparticles exhibited improved in vitro bioactivity and better in vivo bioavailability and effectively eradicated the CAR cells compared to native curcumin [129]. Similarly, curcumin-silk fibroin nanomaterial (Cur-SF) had a stable delivery in HCT116 cells and resulted in a stronger anticancer effect compared to its free-form. It was deduced from this study that the controlled release of CUR-SF enabled its enhanced cellular uptake in HCT116 cells, while the toxicity to normal cells was minimized [42].

Innovation in nanotechnology has highly impacted PDT, as well as other sectors in biomedicine. Nanomaterials can be utilized to improve solubility and deliver and encapsulate the PSs used in PDT. Most PSs, including curcumin, are noted to be hydrophobic-insoluble compounds, with poor bioavailability [96]. Curcumin nanoformulations are recognized to tremendously enhance curcumin′s potential as PS in cancer PDT and are regarded as promising therapeutic modalities for several types of cancer [126,127]. Likewise, higher cytotoxicity can be induced by curcumin if charged on a nanocarrier. Yet, the quality can be amplified via blue-light irradiation exposure, and a greater amount of ROS can be generated to hinder mitochondrial membrane and cell components, causing apoptosis in cancer cells [126]. A study on mouse 4T1 breast cells evaluated the impact of curcumin nanodrugs (Cur-NDs) in combination with PDT. A blue-light source was used with a wavelength of 450 nm and power of 640 mW, and the Cur-NDs preparation was achieved without the use of toxic solvents via the green precipitation and simple method. The treatment of the 4T1 cells was performed in vitro, and the cells were irradiated for 1 min and incubated for 24 h. Considerable increases in cleaved caspase 3, Bax, and p-JNK expressions were affirmed; a large quantity of ROS was produced; MAPKs was activated with apoptosis induced; and cell viability decreased. The study validates Cur-NDs as a valuable PDT photosensitizer that can eradicate breast cancer. The Cur-NDs are thus presented as a good photosensitizer for exploration in clinical practice [130]. Another study examined the effect of curcumin-loaded PEGylated lipid nanomaterial integrated with violet light (410 nm) PDT. Increased cytotoxicity was recorded in human carcinoma cells (A431) in vitro and in mouse skin carcinoma in vivo [131]. The postulate from this study is strengthened by findings affirming that gold nanoparticles and curcumin liposomes integrated with PDT augmented the cytotoxicity in a solid tumor [132]. Experiments showed that curcumin nanoemulsion acted in PDT as a photosensitizing drug, generating an elevated phototoxic effect. Further, the viability for the treated cells was below 5% for cervical carcinoma (Caski and SiHa) cell lines, and the human keratinocytes (HaCa) cell lines were spontaneously immortalized. Caspase 3 and 4 activities were increased, signifying that the death cells arose via apoptosis. The utilization of curcumin nanoemulsion as a drug in PDT via an in situ fiber laser was suggested by the authors as a therapeutic alternative for treating cervical cancer [133]. Table 2 summarizes some recent research outcomes of nanocurcumins in cancer PDT. These innovative treatment approaches can help in overcoming curcumin′s poor solubility and bioavailability, leading to remarkable anticancer effects, such as reducing tumor volume, high cytotoxicity, preclusion of metastasis, massive stimulation of apoptosis, high quantity of ROS generated, and significant damage to mitochondrial membranes in cancerous cells.

## 8. Conclusions

Curcumin is an active natural compound that exhibits therapeutic effects on different diseases, including antiviral, antibacterial, anti-amyloid, thrombo-suppressive, antiarthritic, antioxidation, anti-inflammatory, and anticancer, with minimal aftereffects. Curcumin is safe in humans and has chemopreventive and chemotherapeutic effects. It can be used as a photosensitizer in PDT. In vitro experimental evidence indicates that curcumin has excellent anticancer ability and can target cancer via diverse mechanisms by modulating several cellular signal pathways. Yet, the low oral availability, rapid metabolism, and low cellular uptake associated with curcumin’s poor solubility in water greatly limit its application in cancer therapy.

The incorporation of curcumin as a photosensitizer in cancer PDT can help to surmount some of the challenges associated with poor bioavailability by promoting ROS production and massive apoptosis in cancerous cells. This could, as well, limit the aftereffects associated with conventional anticancer therapy, as the exploration of curcumin in PDT is considered a safe and non-invasive treatment strategy. Moreover, the integration of curcumin nanoformulations in PDT may also ameliorate curcumin’s bioavailability, leading to anticancer effects, such as antiproliferation, high cytotoxicity, reduction in tumor volume, significant apoptosis, mitochondrial membrane damage, and an elevated quantity of ROS generated. Though free curcumin or curcumin in nanoformulations have a remarkable PDT impact, this innovative anticancer treatment strategy seems to be mostly limited to in vitro studies. Extending this treatment strategy to clinical applications in the near future could be very beneficial to many cancer patients since PDT is non-invasive.

## Figures and Tables

**Table 1:** Photophysical properties of curcumin.

Properties	Results	Reference
Excitation Coefficient	In ethanol 27,470 M^−1^ cm^−1^ (430 nm), in toluene 42,760 M^−1^ cm^−1^ (420 nm), and in methanol 55,000 M^−1^cm^−1^ (422 nm).	[34,35]
Maximum absorption (λ max)	In toluene 420 nm, in acetonitrile 422 nm, and in 430 nm each ethanol, methanol, and dimethylsulfoxide (DMSO).	[34]
Solubility	In water at 25 °C and DMSO. Solubility in solvents with acetone > 2-butanone > ethyl acetate > methanol > ethanol.	[36,37]
Photobleaching (%) of 50 μg·mL^−1^ of curcumin	405 nm LED at 5, 15, 45, and 105 min equal to 11, 19, 30, and 45%, respectively.	[38]
Phosphorescence lifetimes at low temperature	7.2 in ethanol.	[34]
Glasses T(Ph) at 77 K ms	11.5 in toluene.	[34]
Yield of quantum singlet oxygen Φ (^1^O_2_)	<0.01 in ethanol, and 0.11 in both acetonitrile and toluene.	[34]
Quantum yield of fluorescence Φ (F)	0.063, 0.067, 0.104, and 0.027 in ethanol, toluene, acetonitrile, and DMSO, respectively.	[34]
Maximum fluorescence position (λ^F^ max)	In toluene 460 and 488, in ethanol 549, in N, N-dimethylformamide 540, and in acetonitrile 524.	[34]

**Table 2:** Some recent research outcome of nanocurcumins in cancer PDT.

Curcumin Nanoformulation	Light Type Source	In Vitro Application	Outcome	References
Curcumin nanoemulsion	LED at 447 ± 10 nm, power 420 mW, fluency 80 J/cm^2^	Cervical cancer (SiHa, and CasKi) cell lines and human keratinocytes (HaCaT) cell lines	Increased activity of caspases 3 and 7 and this indicates cell death occurred via apoptosis.	[133]
Curcumin nano-lipid-carriers (Cur-NLCs)	Blue light (430-nm), fluency 6 J/cm^2^, power 100 mW	Human breast cancer (MCF-7) cells	Strong anticancer activity, increased Cur-NLCs penetrating cancerous cells and elevated cytotoxic effect.	[134]
Curcumin nanoemulsion	Light-emitting diode (LED) at 440 ± 10 nm, power 420 mW, fluency 80 J/cm^2^	Human breast cancer (MCF-7) cells	Phototoxicity with reduction of MCF-7 cell, increased caspases 3 and 7 activity, antiproliferation and the stimulation of ROS.	[135]
Nano PLGA loaded curcumin (Cur-NP)	LED at 457 and 620 nm, fluency 8.6 J/cm^2^	Human ovarian adenocarcinoma (SK-OV-3) cell lines	Cur-NP stable in light and the threshold of curcumin elevated, which led to stronger apoptotic effect on SK-OV-3cells.	[136]
Dendrosomal nanocurcumin	LED at 450 nm, power 75 mW, fluency 0.95 J/cm^2^	Mouse embryonic fibroblast cells (MEFs)	Elevated migration and proliferation of cells, expression of inflammatory cytokines (IL-6, TNF-α) decreased, upregulation of growth factors (VEGF, TGF-β), and elevated intracellular ROS in MEFs.	[137]
Liposomal curcumin (Lip-Cur)	Blue light at 380 to 500 nm, fluency 2.5 J/cm^2^	Squamous cell carcinoma (SCC-25), melanoma (MugMel2), and normal keratinocytes (HaCaT) cell lines.	Stability and bioavailability of lip-Cur, which acted as potent photosensitizer, triggering apoptosis in cancerous cells. Low phototoxic effect was induced on the normal HaCaT cells.	[138]
F127-curcumin micelles	LED 450 nm, power 0.0024 W/cm^2^, fluency 6.3 J/cm^2^	Mouse colorectal carcinoma (CT26) cells	Reduction in cell volume, and the treatment method anticipated as promising for cancer eradication.	[139]
Curcumin hydrogel;curcumin conjugated with silver nanoparticles (Cur-AgNPs)	Blue light at 424 nm, intensity 0.025 W/cm^2^	Human colon (Caco-2) cell lines	Incorporating curcumin with the hydrogels helped in regulating the release Cur-AgNPs in the cells. Cur-AgNPs in PDT led to markedinhibition of Caco-2 cells.	[140]
Nano-encapsulated curcumin conjugatedwith the epidermal growth factor	Blue LED at 460 nm, power 5 ± 0.1 mW, intensity 9 J/cm^2^	Non-cancerous gastric-epithelial mucosa (GHG) human cellsand human gastric cancer (MKN45) cell lines	Encapsulated curcumin with tripolyphosphate/chitosan nanomaterials had superior PDT impact on cancer cells. The method was proposed for tumors that overexpress EGF-R.	[141]
Curcumin solid–liquid nanomaterial (Cur-SLN)	LED at 430 nm, power 0.05 W/cm^2^	Human non-small lung cancer (A549) cells	The Cur-SLN photosensitizer triggered great apoptosis in A549 cells. Expression of caspases 3 and 9 were increased, and the ratio for Bax/Bcl-2 was increased. Intense intracellular shuttling of curcumin, with its improved hydrolytic stability.	[142]
Curcumin-loaded PLGA nanoparticles in the form of nano-in-microparticles	LED at 457 nm, fluency 33.0 and 66.1 J/cm^2^	Human lung cancer (A549) cells	Elevated phototoxicity that varied depending on the radiation dose. The nano-in- microparticles were suggested as effective drug carrier for lung cancer PDT.	[143]

## Data Availability

Not applicable.
